# Pathways to adolescent social anxiety: Testing interactions between neural social reward function and perceived social threat in daily life

**DOI:** 10.1017/S0954579424001068

**Published:** 2024-05-27

**Authors:** Stefanie L. Sequeira, Jennifer S. Silk, Neil P. Jones, Erika E. Forbes, Jamie L. Hanson, Lauren S. Hallion, Cecile D. Ladouceur

**Affiliations:** 1 Department of Psychology, University of Virginia, Charlottesville, VA, USA; 2 Department of Psychology, University of Pittsburgh, Pittsburgh, PA, USA; 3 Department of Psychiatry, University of Pittsburgh, Pittsburgh, PA, USA

**Keywords:** adolescence, ecological momentary assessment, fMRI, peers, social anxiety

## Abstract

Recent theories suggest that for youth highly sensitive to incentives, perceiving more social threat may contribute to social anxiety (SA) symptoms. In 129 girls (ages 11–13) oversampled for shy/fearful temperament, we thus examined how interactions between neural responses to social reward (vs. neutral) cues (measured during anticipation of peer feedback) and perceived social threat in daily peer interactions (measured using ecological momentary assessment) predict SA symptoms two years later. No significant interactions emerged when neural reward function was modeled as a latent factor. Secondary analyses showed that higher perceived social threat was associated with more severe SA symptoms two years later only for girls with higher basolateral amygdala (BLA) activation to social reward cues at baseline. Interaction effects were specific to BLA activation to social reward (not threat) cues, though a main effect of BLA activation to social threat (vs. neutral) cues on SA emerged. Unexpectedly, interactions between social threat and BLA activation to social reward cues also predicted generalized anxiety and depression symptoms two years later, suggesting possible transdiagnostic risk pathways. Perceiving high social threat may be particularly detrimental for youth highly sensitive to reward incentives, potentially due to mediating reward learning processes, though this remains to be tested.

Social anxiety disorder (SAD) is characterized by a persistent fear of being negatively evaluated in social situations, leading to high distress and avoidance of social situations. Rates of SAD increase significantly in adolescence, particularly for girls. By age 18, more than one in 10 girls in the United States will have met diagnostic criteria for SAD (Merikangas et al., 2010). Many more adolescents will experience distressing and functionally impairing symptoms of social anxiety but do not meet full diagnostic criteria for SAD; girls are at high risk for increases in subclinical social anxiety symptoms around ages 14–15 years (Ranta et al., 2007). Social anxiety is associated with academic underachievement, smaller social networks, increased risk for suicide and substance use, and high levels of loneliness, dysphoria, and generalized anxiety (Beidel et al., 1999; Ginsburg et al., 1998; Kashdan & Herbert, 2001). Though efficacious treatments for SAD exist (e.g., Scaini et al., 2016), not all youth respond to such treatments, and treatment gains may not be maintained long (Kerns et al., 2013). Clarifying the pathways through which social anxiety symptoms develop in adolescent girls may help identify new targets for intervention. To this end, the present study examined whether girls with higher neural responsivity to social reward cues are at highest risk for social anxiety symptoms over time when they perceive greater social threat in their peer interactions. This work is informed by recent developmental theories of social anxiety and social anhedonia, which propose that higher social threat contributes to symptoms and correlates of social anxiety in youth more sensitive to incentives due to mediating reinforcement learning processes (Richey et al., 2019; Sequeira et al., 2022).

Social anxiety has often been studied in relation to heightened threat reactivity, and young people with (and at risk for) social anxiety are more prone to perceiving threat in their social interactions (Dapprich et al., 2023; Kingsbury & Coplan, 2016). Emerging research also suggests that aberrant neural reward responsiveness may play a role in the development of social anxiety. Specifically, research has found that youth high in behavioral inhibition (BI), an early-emerging temperament characterized by a fear of novelty (Kagan et al., 1984), show heightened activity in the striatum when viewing reward cues (i.e., stimuli that signal the potential for future reward) and anticipating social evaluative feedback from preferred peers (Bar-Haim et al., 2009; Guyer et al., 2006, 2014). Importantly, high BI in childhood is associated with almost a six-fold increase in odds of developing SAD in adolescence (Sandstrom et al., 2020). High activation to monetary reward cues in the caudate, a region of the striatum, has also been shown to confer risk for future social anxiety symptoms in youth high in BI (Pérez-Edgar et al., 2014). Additionally, Guyer et al. (2012) found that in youth with SAD, caudate activation to incentive cues (i.e., cues signaling potential monetary reward or loss) increased as the magnitude of the reward or loss increased; this pattern of findings was not seen in youth with generalized anxiety disorder. Interestingly, associations between high striatal activity to incentive anticipation and general anxiety symptoms were also not found in a larger community sample of youth not recruited to be high in social anxiety symptoms or BI (Mikita et al., 2016).

Taken together, findings could suggest that high striatal activation to reward cues could be a mechanism specific to the development of social anxiety symptoms and SAD. In the newly proposed Detection and Dual Control (DDC) framework, Fox et al. (2023) propose that in youth high in BI, higher striatal responses to stimuli signaling reward could reflect increased detection processes, or processes involved in registering and orienting attention to potentially important information in the environment. Indeed, prior research suggests that neural activity emerging 300–500 ms post-reward cue relates to attention allocation to reward cues (Broyd et al., 2012; Kohls et al., 2011; Nieuwenhuis et al., 2005; Wang et al., 2020; Wei et al., 2016). Higher neural responses to social reward cues may also support higher approach-related affect and motivation, as recognizing that a cue predicts a potential reward and allocating attention to said cue enhances one’s ability to obtain that reward. Importantly, though, not all youth who are highly neurobiologically or behaviorally response to potential rewards will develop social anxiety. Moreover, increases in neural responsivity to the anticipation of rewards during adolescence are normative, and may support developmentally appropriate increases in reward-seeking and motivation (Ernst et al., 2009; Forbes & Goodman, 2014; Gilbert, 2012; Paus, 2005; Steinberg, 2008; van Duijvenvoorde et al., 2016).

Several developmental theories suggest that it is the *interaction* between threat- and reward-related factors that confers risk for social anxiety in adolescence. For example, approach-avoidance conflict models (Barker et al., 2019; Caouette & Guyer, 2014; Helfinstein et al., 2012) suggest that high BI and social anxiety are characterized by heightened activity in both the behavioral inhibition system (BIS) and behavioral activation system (BAS), and conflict between these systems. For example, a fearful child entering a novel social environment, such as a school dance, may feel highly motivated to seek out positive social experiences with peers (high BAS) but a co-occurring fear of embarrassment fuels the avoidance motivational system (high BIS). In their DDC framework, Fox et al. (2023) also propose that factors like parenting may impact threat systems that interact with increased detection processes to confer risk for social anxiety in high BI youth.

Additional recent theory and frameworks from Richey et al. (2019) and Sequeira et al. (2022) expand on how high neural reactivity during reward anticipation may interact with social threat to contribute to social anxiety symptoms in adolescents through altered reinforcement learning mechanisms. Higher neural responses to reward cues supporting greater attention to reward-related contingencies in the environment and the consequences of one’s actions (Caouette & Guyer, 2014) may facilitate learning. In some contexts, heightened attention to reward cues may be beneficial; when youth highly attentive to potential rewards repeatedly perceive more rewards, they may learn over time to expect rewards. However, when youth highly attentive to potential rewards repeatedly perceive more punishment, including more social threat, they may come to associate situations that have the potential to be rewarding with threat or punishment, contributing to more severe social anxiety symptoms and social avoidance (see Richey et al., 2019). Thus, interactions between high neurobiological sensitivity to social reward cues and higher perceived social threat may be key for understanding how social anxiety develops in adolescence. This may be particularly true for girls at temperamental risk for social anxiety. Girls are highly sensitive to interpersonal stress (Rudolph, 2002) and shy, fearful youth are more likely to show high neural responses to social reward anticipation and perceive more social threat. For example, consider a shy teen who joins the school musical expecting to receive positive feedback from peers when they get on stage. When the teen gets on stage each day for rehearsal, though, they perceive several peers to be laughing at them. Over time, this teen may come to expect negative social feedback when they get on stage, thus the stage (originally a cue associated with potential reward) comes to be associated with threat or failure and the teen avoids rehearsal. In short, at-risk girls with heightened neural responsivity to social reward cues or contingencies may be at highest risk for social anxiety when they also perceive more social punishment due to modified reinforcement learning processes (Richey et al., 2019; Sequeira et al., 2022).

The interaction between high neural responses to reward cues and perceived threat from *peers* may be particularly important for understanding increases in symptoms of social anxiety. Youth high in shyness or social anxiety report more negative, and less positive, peer relationships (Erath et al., 2007; Ginsburg et al., 1998). Further, peer stress (but not family stress) has been associated with increases in social anxiety symptoms over time in adolescents (Epkins & Heckler, 2011; Griffith et al., 2020). One limitation of the literature linking peer threat to social anxiety, however, is the predominance of one-time questionnaire measures that rely on adolescents remembering their peer relationships from weeks, months, or even years prior. This is subject to retrospective biases and may be influenced by an adolescent’s current functioning (e.g., adolescents who are more socially anxious may report that their interactions over the past month were worse than they felt in the moment). This highlights a need for more fine-grained, state-based measures of adolescents’ perceptions of their peer relationships. Ecological momentary assessment (EMA) is one such approach to studying adolescents’ daily perceptions of their social relationships. Collecting repeated measurements over a span of days and weeks using EMA also allows for a more stable measure of functioning, with minimal retrospective recall bias.

To our knowledge, only one empirical study has tested how interactions between neural reward function and perceptions of peer threat are related to social anxiety symptoms in youth. In a sample of 47 youth (age 11), Jarcho et al. (2019) found that for youth higher in early childhood wariness, higher amygdala activation to the receipt of unpredictable positive feedback was associated with more severe social anxiety symptoms when these youth also reported higher peer victimization. This study provides important preliminary evidence that interactions between neural responses to social reward and perceived peer threat are associated with social anxiety in adolescence. However, the small sample, cross-sectional data collection, and limited assessment of peer threat (one four-item questionnaire assessing peer victimization) support replication in larger, longitudinal samples. How interactions between neural reward function and perceptions of peer threat predict the development of social anxiety symptoms over time remains unknown. Additionally, the authors only tested symptoms of social anxiety as the outcome; whether interactions between neural reward function and perceptions of peer threat are specific to the development of social anxiety symptoms or generalize to other anxiety symptoms or symptoms of depression, which frequently co-occur with symptoms of social anxiety, is critical to test.

To address previous limitations and advance understanding of the developmental pathways through which social anxiety symptoms may develop in adolescence, the present study tested interactions between neural responsivity to social reward (vs. neutral) cues and perceptions of social threat in early adolescence (ages 11–13 years) on social anxiety symptom severity in mid-adolescence (ages 13–15 years). We recruited 129 early adolescent girls oversampled for shy or fearful temperament and employed a multimethod, longitudinal design using EMA to assess youths’ perceptions of social threat from peers in daily life at baseline (Wave 1). Of note, while self-report via EMA cannot provide an “objective” assessment of interpersonal peer victimization, it can index how much threat girls perceive in their negative peer interactions. Such perceptions could vary in nature and severity, and girls high in social anxiety may be particularly likely to perceive more social threat. Moreover, the *perception* of higher social threat may be key for influencing reinforcement learning systems.

To measure neural reactivity to social reward cues, we administered a peer-observation version of the Social Incentive Delay task (Cremers et al., 2015) in the MRI scanner at baseline (Wave 1). During the anticipation phase of this Peer-Social Incentive Delay (P-SID) task, participants view different shapes (cues) that alert them to the types of performance-related feedback they can receive from “peers” they believe to be observing them complete a task; this P-SID task is highly developmentally relevant given the salience of peer evaluative feedback in adolescence. We measured neural activity during the presentation of cues that signal potential positive peer feedback (i.e., social reward cues) versus cues that signal definite neutral peer feedback (i.e., neutral cues) in several regions-of-interest (ROIs), or regions of the brain that play a role in social reward processing, including the caudate head, caudate body, putamen, NAcc, anterior insula (AI), basolateral amygdala (BLA), precuneus, dorsal ACC, and mediodorsal nucleus (MDN) of the thalamus (Martins et al., 2021; Rademacher et al., 2010). At baseline and two years later (Wave 2), social anxiety symptoms were assessed by clinical interviewers and youth self-reported on their symptoms of generalized anxiety and depression.

Our primary analytic approach was to apply factor analysis to identify potential latent factors associated with social reward neural function. Latent variable approaches have been used to model brain structure and function in prior work (e.g., Baskin-Sommers et al., 2016; Bolt et al., 2018; Kim-Spoon et al., 2021; Kurkela et al., 2022; Lahey et al., 2012). There are many benefits of creating a latent factor to measure brain function, including improvements in reliability (Cooper et al., 2019; Kim-Spoon et al., 2021), though this approach may sacrifice specificity that comes with testing individual ROIs in separate models. Resulting factors were included in structural equation models to test how neural social reward function interacts with daily perceptions of social threat to predict social anxiety symptoms.

Aligning with prior theory (Richey et al., 2019; Sequeira et al., 2022), we hypothesized that girls perceiving higher social threat in daily peer interactions would exhibit the greatest social anxiety symptom severity two years later when they showed high neural responses to cues signaling potential positive feedback from peers (i.e., social reward cues) relative to cues signaling neutral peer feedback (i.e., neutral cues) at baseline. We also hypothesized that when girls perceived very low levels of social threat from peers, they would exhibit the lowest social anxiety symptom severity over time when they also showed high neural responses to social reward (vs. neutral) cues. These hypotheses align with a neurobiological susceptibility to social context model, which is based in differential susceptibility theory and proposes that youth with certain neurobiological factors are more responsive to their social contexts for better and for worse (Schriber & Guyer, 2016). We also consider these hypotheses through the lens of social reinforcement learning. As previously described, high neural reactivity to social reward cues could promote learning from social rewards in youths’ environments, and this learning may be affected by the perception of high social threat. In environments high in perceived social threat, youth high in neurobiological responsivity to potential social rewards may come to associate potential rewards with social threat or failure, laying the groundwork for fear of negative evaluation and related social anxiety symptoms. In environments low in perceived social threat, youth high in neurobiological responsivity to social rewards cues may be more likely to learn from (and come to expect) socially rewarding feedback, which may be protective against increase in social anxiety symptoms.

Importantly, we examined specificity of this model to social anxiety (versus generalized anxiety and depression) and social reward (versus punishment) using a series of sensitivity analyses. We hypothesized specificity to social anxiety, as most prior research has linked depression symptoms (and to a lesser extent generalized anxiety symptoms) to reduced neural and behavioral reactivity to rewards (Forbes & Dahl, 2012; Keren et al., 2018). However, we hypothesized that the model would generalize to neural activation to punishment cues. High striatal activity to cues signaling potential monetary losses has also been linked to high BI (Guyer et al., 2006), potentially because loss cues are also highly motivationally salient. High striatal activation to reward *or* loss cues could signal heightened attention towards incentive contingencies, which may facilitate learning. Though we focused the study on neural reactivity to social *reward* cues, youth who are more neurobiologically responsive to social punishment may also be more likely to attend to and learn from social threat in their environment, contributing to more severe social anxiety symptoms over time.

## Methods

### Participants

One-hundred-twenty-nine early adolescent girls ages 11–13 were recruited for participation in a longitudinal study via online advertisements and announcements in the community. See Table 1 for demographic and clinical characteristics. Girls were recruited based on parent-reported sex at birth; gender identity was not assessed. We oversampled for shy/fearful temperament, a risk factor for future social anxiety disorder (Sandstrom et al., 2020). Temperament was assessed using the Early Adolescent Temperament Questionnaire-Revised (EATQ-R; Ellis & Rothbart, 2001), which was designed to measure temperament traits in early adolescence (ages 9–15), with items specific to adolescent life experiences. To determine temperament status, participants were compared against established distribution scores of the EATQ-R shyness and fear scales (Ellis & Rothbart, 2001). The sample was stratified such that approximately 2/3 of participants (*n* = 85) scored > 0.75 SDs above the mean on the parent- or adolescent-rated fear scales (3.12 for parent-report, 3.48 for adolescent-report) or shyness scales (2.99 for parent-report, 3.16 for adolescent-report). All other participants (*n* = 44) scored below this cutoff and were in the normative range of shy/fearful temperament.

Exclusionary criteria included a current or lifetime DSM-5 diagnosis of any anxiety disorder (except specific phobia), obsessive-compulsive disorder, post-traumatic stress disorder, major depressive disorder, or any psychotic or autism spectrum disorder, as determined by the Kiddie-Schedule for Affective Disorders and Schizophrenia (K-SADS-PL; Kaufman et al., 1997). Additional exclusionary criteria included IQ < 70 as assessed using the Wechsler Abbreviated Scale of Intelligence (WASI; Wechsler, 2011), lifetime presence of a neurological or serious medical condition, presence of any MRI contraindications, presence of head injury or congenital neurological anomalies (based on parent report), acute suicidality, medications that affect the central nervous system, and ocular conditions that would impede eye tracking measurement and/or ability to see clearly without prescription glasses. Stimulants were permitted if use was discontinued for 36 hours prior to the scan.

All participants were included in analyses; missing data were estimated using full-information maximum likelihood procedures. For completeness, though, we report reasons for missing data for the primary variables in the model. First, fMRI data were available for 87 participants. Reasons for missing fMRI data included: (1) Excess movement (*N* = 25), (2) Scan not completed (*N* = 9), (3) Task data not collected during scan due to scanner or task error (*N* = 7), or (4) An incidental finding that impeded analyses (*N* = 1). Usable EMA data were available for 105 participants. EMA data were missing due to: (1) Low completion rates (< 25%; *N* = 4), (2) Data quality issue (i.e., random responding; *N* = 2), (3) Less than 3 negative interactions with peers (*N* = 11), (4) EMA dropout or study withdrawal (*N* = 6), or (5) Technical problem (no data collected; *N* = 1). Wave 1 social anxiety symptom data were available for 126 girls; three girls had missing data due to issues with administration (i.e., questionnaire administered incorrectly by study diagnostician). Wave 2 social anxiety symptom data were available for 117 girls; 12 girls had missing data because they dropped out from the study prior to data collection. Girls with missing data did not significantly differ from girls with full data on any of the measures included in the final analysis (*ps* > .10). Girls missing EMA data but not fMRI data (*N* = 15) differed from girls missing both EMA and fMRI (*N* = 9) in pubertal status (*t*(22) = 2.5, *p* = .020), such that girls missing both measures were less advanced in pubertal status. No differences in age, risk type, social anxiety symptoms, generalized anxiety symptoms, or depressive symptoms were seen between girls missing fMRI but not EMA (*N* = 33), girls missing EMA only, and girls missing both fMRI and EMA. Complete data (i.e., usable EMA, fMRI, and social anxiety symptom data) were available for 68 participants.

### Procedure

The study was approved by the University of Pittsburgh Institutional Review Board. Parents provided informed consent and youth provided informed assent to acknowledge their voluntary agreement to participant in the research. Data were collected from multiple laboratory visits conducted over a three-year period between 2016 and 2021. Following informed consent (Wave 1), a research assistant administered the WASI and a clinical interviewer (a master’s level graduate student or doctoral level therapist) administered the K-SADS-PL to determine eligibility and the Liebowitz Social Anxiety Scale for Children and Adolescents (LSAS-CA; Masia et al., 1999) for a measure of adolescent social anxiety symptoms. During a follow-up visit to the lab, approximately two weeks after the initial visit, participants were given an android smartphone to complete an EMA home protocol. Approximately two weeks later, youth completed the Peer Social Incentive Delay (P-SID) task at the University of Pittsburgh Magnetic Resonance Research Center. Approximately two years after the initial visit (Wave 2), the LSAS-CA was readministered to measure social anxiety symptoms.

#### Ecological momentary assessment (EMA) protocol

Data on real-world social threat experiences were collected using cell-phone EMA at Wave 1. Youth were given a preprogramed android smartphone on which they entered responses to a series of questions about their daily experiences with peers using a secure smartphone app for Web Data Express developed by the Office of Academic Computing in the University of Pittsburgh Department of Psychiatry. Using these phones, participants were asked to answer questions about their most recent social interactions and their emotional responses to these interactions for 16 consecutive days. Adolescents were randomly sampled (i.e., received an electronic notification to respond) three times per day on weekdays (once in the morning between 7 AM and 8 AM and twice between 4 PM and 9:30 PM) and four times per day on the weekends between 10 AM and 9:30 PM, allowing for a maximum of 54 observations. This large number of samples allows for a more stable estimate of “typical functioning,” even in the potential presence of several atypical days. Compliance in this sample was 81.3% (SD = 13.9%, range = 37.0%–100%).

#### fMRI acquisition

Before entering the real MRI scanner at both Wave 1 and Wave 2, participants were trained in a simulation MRI scanner (“mock scanner”) to familiarize them to the tight space and the loud sounds of the scanner. They were also instructed about how to keep still during the scan to prevent motion artifact. Scanning took place on the same 3T Siemens Prisma magnet at both time points. Task stimuli were projected onto a color, high-resolution LCD screen in front of the scanner bed and viewed in a mirror mounted on the head coil. Head movement was constrained by foam padding. Participants responded to stimuli using a handheld response glove on their right hands; all participants were right-handed.

Anatomical images covering the entire brain were acquired first using a three-dimension magnetization-prepared rapid gradient-echo T1-weighted sequence (repetition time [TR] = 2300 ms, echo time [TE] = 3.93 ms, flip angle 9°, inversion time [TI] = 900 ms, voxel size = 1 mm^3^). Functional scans were preceded by a localizer. Functional images were acquired using multiband gradient echoplanar sequences (60 slices, three-factor multiband) sensitive to BOLD contrast [T2*] (TR = 1500 ms, TE = 30 ms, flip angle 55°, voxel size = 2.3 × 2.3 × 2.3 mm). Field maps were acquired using gradient echo planar imaging sequence for correction of field distortions in the functional images with the following parameters: TR = 590 ms, TE1 = 4.92 ms, TE2 = 7.38 ms, voxel size = 2.3 × 2.3 × 2.3 mm, flip angle 60°. Following this scan, the Chatroom Interact Task (Silk et al., 2012) was first administered, followed by the Peer Social Incentive Delay (P-SID) task.

### Measures

#### Social anxiety symptoms

The Liebowitz Social Anxiety Scale for Children and Adolescents (LSAS-CA; Masia et al., 1999) is a clinician rating scale used to measure social anxiety symptoms in youth. The measure consists of 24 items, 12 social interaction situations (e.g., “looking at people you don’t know well in the eyes”) and 12 performance situations (e.g., “asking questions in class”). The clinician reads each social situation to the adolescent and their participating parent and asks the adolescent to rate how anxious each situation made them over the past week on a Likert scale of 0 (not at all) to 3 (very much). The adolescent is also asked to rate how much they tried to avoid the situation using the same 0 to 3 scale. Parents are asked to provide their input, and the clinician can adjust the adolescent’s ratings based on parent input, clinical judgment, and direct behavioral observations. The total LSAS-CA score (integrating ratings of anxiety and avoidance in social situations) was used for the present study (baseline: *α* = .94; two-year follow-up: *α* = .96).

#### Social threat EMA measure

This measure has been used and validation in this sample in prior work (see Sequeira et al., 2021). To assess youths’ perceptions of social threat in daily life, the following prompt was provided at each observation: “Think about the interaction with other kids your age that made you feel the worst since the last beep.” Participants were asked to type out details about this interaction, which were reviewed during data cleaning. If participants could not think of a negative interaction, they received further probes to help them think of an event. If participants continued to indicate that they did not have a negative interaction, this observation was coded as “no negative interaction” and coded as missing data. Participants were then given a checklist of statements that described how they may have been thinking or feeling during the interaction (referred to as “social threat statements”) and asked to check off which statements applied to them in the situation. Examples of social threat statements include, “I felt criticized,” and “I felt disliked or rejected” (see Sequeira et al., 2021 for full details).

#### Peer social incentive delay (P-SID) task

The Peer Social Incentive Delay (P-SID) task (Hutchinson et al., 2021; Kaurin et al., 2022) is a peer-observation adaptation of the original Social Incentive Delay task (Cremers et al., 2015) designed to measure brain activity related to social rewards and punishments. This “peer observation” version of the task was created to examine neural activation to social feedback from a virtual peer. At a laboratory visit prior to the scan, participants viewed fictional photos and autobiographical profiles (including hobbies and personality traits) of age-matched girls whom they were told were participating in the study at other institutions. Participants were asked to select and rank which girls they would most like to interact with during the MRI scan.

At the start of the fMRI visit, participants were told that two of the girls they ranked most highly would be interacting with the participant from other sites during the fMRI tasks. Further, they were told that one girl would be watching the participant complete the P-SID task and providing feedback after each trial by sending a smiling, frowning, or neutral (blurry) picture of themselves based on the participant’s performance. To increase believability, participants were also asked to view (via a mock video feed) and evaluate this peer’s performance on the P-SID task prior to their own scan. The peer’s performance on the P-SID task was computer-generated. Following the task, participants were debriefed about the social deception.

The P-SID task consists of one run of 72 trials (27 social reward, 27 social punishment, 18 control). Each trial proceeds in the following order: presentation of a cue (500 ms), fixation cross (1500–3500 ms), target (160–500 ms), black screen (1000 ms), virtual peer feedback (1650 ms) and black screen (2500–5000 ms). Participants were instructed to press a button with their index finger as quickly as possible when the target (a filled in white square) appeared on the screen. The target slide was always presented for 500 ms but target presentation on that slide was variable (160–500 ms) to ensure that hit rates in different conditions were similar across participants. Total duration of the task was 12 min and 2s (480 volumes).

At the start of each trial, a cue (circle, square, or triangle) signaled the possible outcomes the participant could receive from the virtual peer that reflected the three conditions of the task (social reward, social threat, neutral). In the social reward condition, a circle signaled possible positive feedback (peer’s happy face) for a response that fell within the range of the target presentation (i.e., “hit”) or neutral feedback (peer’s scrambled face) for a response that fell outside the range (i.e., too fast or too slow; “miss”). In the social punishment condition, a square signaled negative feedback (peer’s angry face) for a response that fell outside of target range (i.e., “miss”) or neutral feedback (peer’s scrambled face) for a response within range (i.e., “hit”). In the control condition, a triangle cued a neutral outcome (peer’s scrambled face) regardless of performance. The primary analysis examined neural activity during the presentation of social reward (circle) cues relative to neutral (triangle) cues. Sensitivity analyses examined neural activity during social punishment (vs. neutral) cues.

#### Pubertal status

Pubertal status was assessed using the Female Pubertal Development Scale (PDS; Petersen et al., 1988), a self-report measure of physical development for youth under the age of 16. Shirtcliff et al. (2009) developed a coding system to convert the PDS to a 5-point scale to parallel the physical exam Tanner stages, which was used as a covariate in analyses.

### Measures used for sensitivity analyses

#### Generalized anxiety symptoms

At each wave, participants completed a modified (44-item) version of the Screen for Anxiety and Related Emotional Disorders-Child version (SCARED; Birmaher et al., 1997). The SCARED is a self-report checklist that assesses multiple symptoms of anxiety across several domains of anxiety; sensitivity analyses used the generalized anxiety subscale score. The generalized anxiety subscale includes nine items (e.g., “I am a worrier,” “People tell me that I worry too much”). This subscale demonstrated acceptable internal consistency at baseline (***α*** = .82) and two-year follow-up (***α*** = .81).

#### Depressive symptoms

At each wave, participants completed the 33-item Mood and Feelings Questionnaire (MFQ)-Child Version (Angold & Costello, 1987) to assess depressive symptoms over the past two-week period. Each item on the MFQ is rated on a three-point scale (0 = not true, 1 = sometimes true, 2 = true) and summed to create a total score. Scores range from 0 to 66, with higher scores indicating greater depressive symptoms. In the present sample, the MFQ demonstrated high internal consistency at baseline (***α*** = .92) and two-year follow-up (***α*** = .94).

### Analytic plan

#### fMRI data preprocessing and analysis

Statistical Parametric Mapping software (SPM12; Wellcome Trust Centre for Neuroimaging, UK) was used to preprocess functional images. Preprocessing included: (1) Reorientation of anatomical and functional images to the anterior and posterior commissure line, (2) Use of the FieldMap toolbox to create a voxel displacement map (VDM) for distortion correction of the functional images, (3) Use of the Realign and Unwarp procedure to generate motion parameter files and correct for distortion using the VDM, (4) Registration of functional images to the anatomical image, (5) Segmentation of anatomical images into gray and white matter maps using the International Consortium for Human Brain Mapping tissue probability maps, (6) Registration of anatomical and functional images to MNI space using the ICBM152 template with 2 mm voxels, (7) Smoothing of normalized images using a 6 mm^3^ full-width at half-maximum gaussian kernel, and (8) Repair of motion artifacts using ArtRepair (Mazaika et al., 2007). Scans with > 0.5 mm of incremental motion, > 3 mm from the baseline image, and/or 3 standard deviations [SD] intensity shifts were considered outliers; outlier scans were replaced with a linear interpolation between the two nearest non-outlier scans. Participants with > 25% of volumes with excess movement (i.e., outliers) were excluded.

For the first-level analyses, individual effects were estimated using the general linear model approach implemented in SPM12. The following task conditions were modeled at the first level: cue presentation (i.e., presentation of social reward, threat, or neutral cues), social reward feedback hit (i.e., smiling face for hit in the reward condition), social reward feedback miss (i.e., scrambled face for miss in the reward condition), social punishment feedback hit (i.e., scrambled face for hit in the punishment condition), social punishment feedback miss (i.e., angry face for miss in the punishment condition), and neutral feedback (i.e., scrambled face in neutral condition), with motion parameters included as nuisance regressors. Fixation crosses, black screens, and targets were not modeled and thus included in the baseline. Similar to prior research assessing neural reward function in similar samples of youth with or at-risk for social anxiety disorder (e.g., Guyer et al., 2012, 2014; Pérez-Edgar et al., 2014), we focused analyses on neural activity during the social reward, punishment, and neutral cues, with the main contrasts of interest defined as social reward cue > neutral cue and social punishment cue > neutral cue.

**Figure 1.**
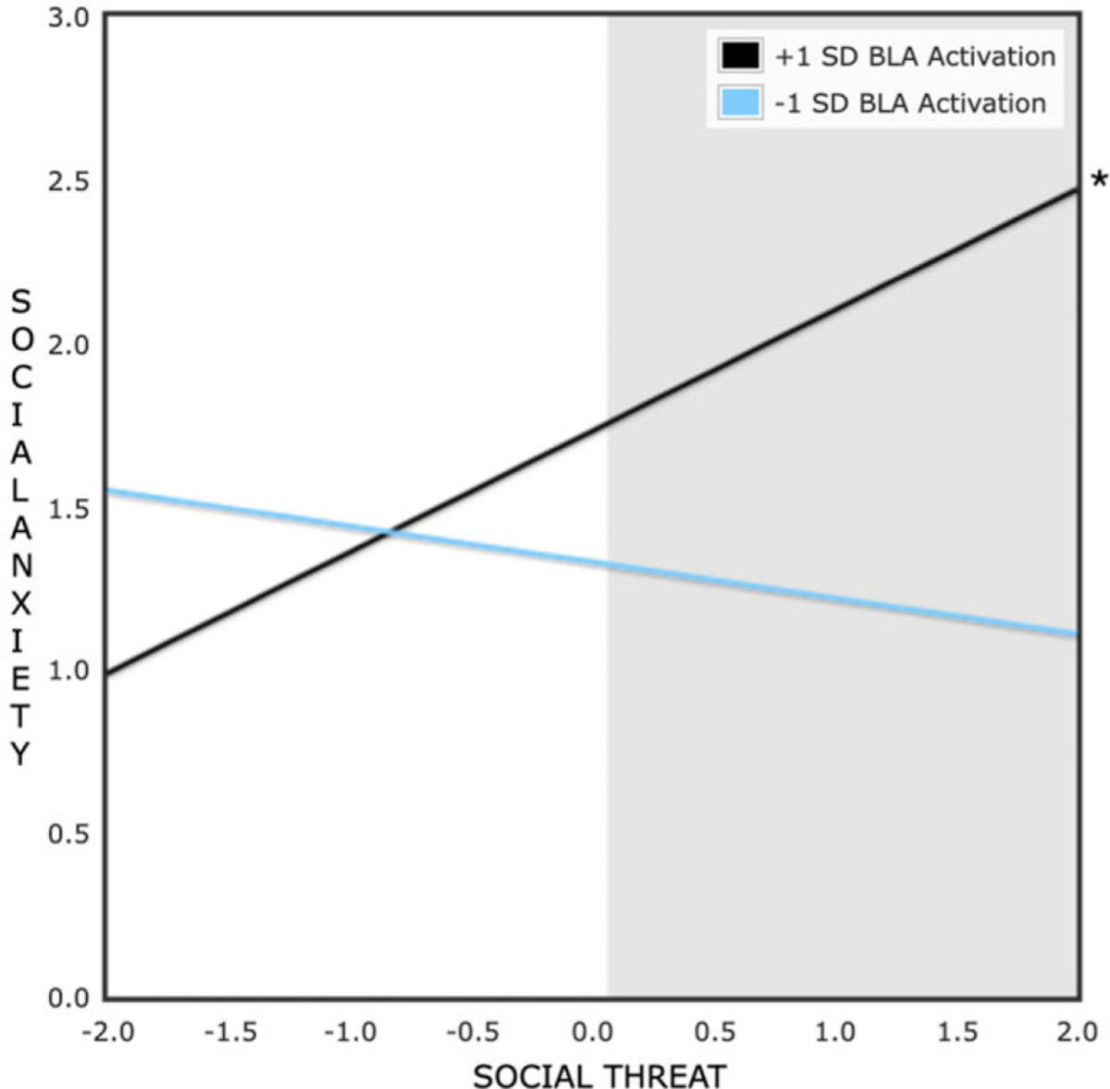
Effect of daily social threat perceptions on two-year follow-up social anxiety symptoms at high (+1 SD) and low (−1 SD) levels of bilateral basolateral amygdala activation to social reward (vs. neutral) cues at baseline. Predictors were standardized prior to the formation of this plot; social threat scores are plotted from −2 SD to 2 SD with a mean of 0. *p < .05.

Group-level analyses focused on several ROIs: the caudate head, caudate body, putamen, NAcc, anterior insula (AI), basolateral amygdala (BLA), precuneus, dorsal ACC, and MDN of the thalamus. Average parameter estimates (mean activation over the entire region) were extracted from each ROI for the contrasts of interest (i.e., social reward cue > neutral cue; social punishment cue > neutral cue) using the MarsBar toolbox for SPM12. The decision to focus on these ROIs is informed by prior research on neural social reward function in adolescents (Martins et al., 2021; Rademacher et al., 2010). The dACC, putamen, AI, and precuneus ROIs were constructed using the Brainnetome Atlas (http://www.brainnetome.org/). The caudate body, caudate head, and mediodorsal nucleus of the thalamus were defined using the Talaraich atlas in WFU Pick Atlas, and the Nacc was defined using the IBASPM71 atlas in Pick Atlas. The BLA was defined in Pick Atlas as a 3.5 mm sphere centered at (*x* = −26, *y* = −5, *z* = −23 for left BLA; *x* = 29,*y* = −3, *z* = −23 for right BLA), as in previous studies on these region (e.g., Gao et al., 2021). All ROIs were bilateral; unilateral ROIs were combined using the FSL -maths function. ROIs are displayed in the online supplement (Figure 1S).

#### Social threat EMA analysis

Social threat sum scores (i.e., the sum of the social threat statements endorsed for each negative peer experience) were used in analyses. Items were summed across each observation because we assumed each item to be weighted equally. Previous research using multilevel exploratory factor analysis has shown that these eight social threat items load on a one factor solution at both the within- and between-person level (Sequeira et al., 2021). These social threat scores can be aggregated across time to create one measure of average social threat for each participant; previous research has shown that this is a reliable and valid measure of social threat (Sequeira et al., 2021).

### Final analytic plan

IBM SPSS Version 26 was used to evaluate descriptive statistics for observed variables, changes in anxiety symptoms over time, and correlations between observed variables. The remaining analyses were conducted using Mplus version 7 (Muthén & Muthén, 1998–2015). First, as a data reduction technique, an exploratory factor analysis (EFA) was run with all ROIs to examine the factor structure of neural social reward function. An EFA was chosen because several different but equally plausible and theoretically sound results were deemed possible. First, all regions could load significantly on one factor of social reward cue responsivity. A second possibility was that two factors may arise: one “reward” factor potentially consisting of the caudate, putamen, Nacc, amygdala, and thalamus and one “social salience” factor potentially consisting of the AI, dorsal ACC, and precuneus. A third possibility was that three or more factors would arise (or some regions would fail to load on a factor). Should multiple factors arise (or some regions fail to load on a factor), we decided a priori to test the most theoretically sound factor(s) first, with additional factors (or regions that did not load on a factor) tested in secondary models. Factor loadings of .40 and above were considered significant; modification indices were considered to improve model fit. As in previous work (e.g., Baskin-Sommers et al., 2016; Oshri et al., 2019), the factor structure that was deemed the best solution was then subject to a confirmatory factor analysis (CFA).

The structural model (including the latent factor) was estimated using structural equation modeling (SEM) in Mplus. The model was estimated using full-information maximum likelihood (FIML; Enders & Bandalos, 2001) and the robust maximum likelihood (MLR) estimator, which features robust standard errors. The model was first tested without the interaction term to determine model fit. Five fit statistics were used to evaluate overall fit of the measurement and structural models: the chi-square (*χ*^2^) statistic, Comparative Fit Index (CFI), the Tucker-Lewis Index (TLI), the Root Mean Square Error of Approximation (RMSEA), and the Standardized Root Mean Square Residual (SRMR). Conventional cutoff criteria proposed by Hu and Bentler (1999) were used to assess model fit: RMSEA < 0.06, CFI > 0.95, TLI > 0.95, and SRMR < 0.08. The *χ*^2^ tests the hypothesis that the hypothesized covariance matrix differs from the observed covariance matrix. Thus, a non-significant *χ*^2^ is indicative of good model fit. Because absolute fit indices are not estimated when a random slope is added to the model (as is done when estimating an interaction with a latent variable), relative fit indices (i.e., Bayesian information criterion, BIC) and the Satorra & Bentler (2001) scaled chi-square different test (using loglikelihood values) were used to compare models with and without the interaction term (i.e., the interaction between the latent neural social reward factor and daily social threat).

SEM was used to test how interactions between heightened neural reactivity to social rewards (Wave 1) and experiences of social threat (Wave 1) in early adolescent girls oversampled for shy or fearful temperament contribute to social anxiety symptoms in mid-adolescence (Wave 2). Social anxiety symptoms at Wave 1 were covaried on Wave 2 symptoms. The total number of negative interactions with peers was also covaried on Wave 2 symptoms to isolate how the *quality* of negative peer interactions contributes to social anxiety symptoms through interactions with reward brain function, above and beyond the *quantity* of negative interactions. Pubertal status at Wave 1 was covaried on both Wave 2 social anxiety symptoms and the neural social reward latent factor. Pubertal status was measured using the PDS (Petersen et al., 1988; Shirtcliff et al., 2009).

Significant interactions were probed using simple slopes analysis in Mplus. The Johnson-Neyman technique was used to compute regions of significance for the moderator. As we originally hypothesized a crossover interaction aligning with the neurobiological susceptibility to social context hypothesis (Schriber & Guyer, 2016), we further probed significant interactions to determine whether findings align with a differential susceptibility model (Belsky et al., 2007). Specifically, for significant interactions, tests of the regions of significance on X (the predictor) were tested. As per Roisman et al. (2012), the test of the regions of significance on X (RoS on X) determines the range of the predictor variable for which the moderator and the outcome variable are significantly associated with each other (typically bounded by ±/− 2 SD from the mean of the predictor variable). Results are consistent with a differential susceptibility hypothesis if the association between the moderator (neural activity) and outcome variable (social anxiety symptoms) is significant at both high and low ends of the range of the predictor variable (i.e., within ±/− 2 SDs of daily social threat). To provide additional support for a differential susceptibility model, Roisman et al. (2012) also suggest reporting the proportion of the interaction (PoI) index, which represents the proportion of the total interaction that is represented on the left and right of the crossover point. PoI values between .40 and .60 (ideally near .50) are consistent with a differential susceptibility model. To plot the interactions and generate RoS on X and PoI values, a web-based application developed by R. Chris Fraley was used (https://www.yourpersonality.net/interaction/). Predictor variables were standardized prior to creation of interaction plots using this application to aid in interpretability.

A series of sensitivity analyses were also run to test specificity of the model. We tested how interactions between neural social reward function and daily social threat predict symptoms of generalized anxiety or depression. The model was also run replacing neural activity to social reward cues with neural activity to social punishment cues.

## Results

### Preliminary results

Descriptive statistics and intercorrelations between observed variables, calculated using SPSS version 26, can be found in the online supplement (Table 1S). In the full sample with complete symptom data (*N* = 114), total social anxiety severity increased significantly from Wave 1 to Wave 2 (*t*(113) = 2.10, *p* = .038). Depressive symptoms did not significantly increase across the sample from Wave 1 to Wave 2 (*t*(116) = 1.44, *p* = .152). Generalized anxiety symptoms did increase significantly from Wave 1 to Wave 2 (*t*(113) = 2.35, *p* = .021).

Social anxiety severity at Wave 2 (two-year follow-up) was measured during the COVID-19 pandemic (March 2020 – December 2020) for 20% of the sample (*N* = 26); for the remainder of the sample, Wave 2 symptom severity was measured prior to the COVID-19 pandemic. Social anxiety severity at Wave 2 did not significantly differ (Welch *t*(1, 38) = 3.00, *p* = .092) between girls with symptoms measured during the COVID-19 pandemic (*M* = 36.04, SD = 25.04) and girls with two-year symptoms measured pre-pandemic (M = 26.41, SD = 23.12). Social anxiety symptom severity increased from baseline to follow-up in both groups, though this increase was not significant in either group, likely due to a decrease in power from splitting the sample (pre-pandemic group: *t*(87) = 1.94, *p* = .055, pandemic group: *t*(25) = .84, *p* = .407). These groups (girls with Wave 2 symptoms measured pre-pandemic or during the pandemic) did not differ in age, pubertal status, or risk type (*ps* > .60).

Youth high in shy/fearful temperament did not differ from youth low to moderate in shy/fearful temperament in neural activity to social reward vs. neutral cues in any ROI (*ps* > .25). These temperament groups also did not show differences in daily social threat (*F*(1,104) = .01, *p* = .911). As expected, these groups differed in social anxiety symptom severity at baseline (*F*(1,125) = 17.85, *p* < .001) and at two-year follow-up (*F*(1,116) = 6.40, *p* = .013), such that youth recruited to be higher in shy/fearful temperament had higher social anxiety severity at both time points.

### Neural social reward factor

Eighty-seven participants had usable fMRI data and were included in the EFA. With nine variables included in the model, the minimum amount of data recommended for factor analysis (10 participants per variable) fell just short. The decision to run an EFA and estimate a structural model with the social reward factor was made *a priori* and before confirmation of final sample size. We acknowledge the ongoing debate around the extent to which researchers should follow a pre-specified data analytic plan. Despite unexpected data loss, we believed it worthwhile to maintain our pre-specified plan. We present the following findings as tentative and urge caution when interpreting the results. Additional detail regarding the EFA is provided in the online supplement. Briefly, all regions except for the basolateral amygdala (BLA) loaded significantly on one social reward factor after making suggested and theoretically sound modifications by adding correlations between the two regions of the caudate and between the dACC and AI. Model fit for this final neural social reward factor was good (*χ*^2^ = 25.54, *df* = 18, *p* = .111; RMSEA = .069, CFI = .99, TLI = .98, SRMR = .038).

**Figure 2.**
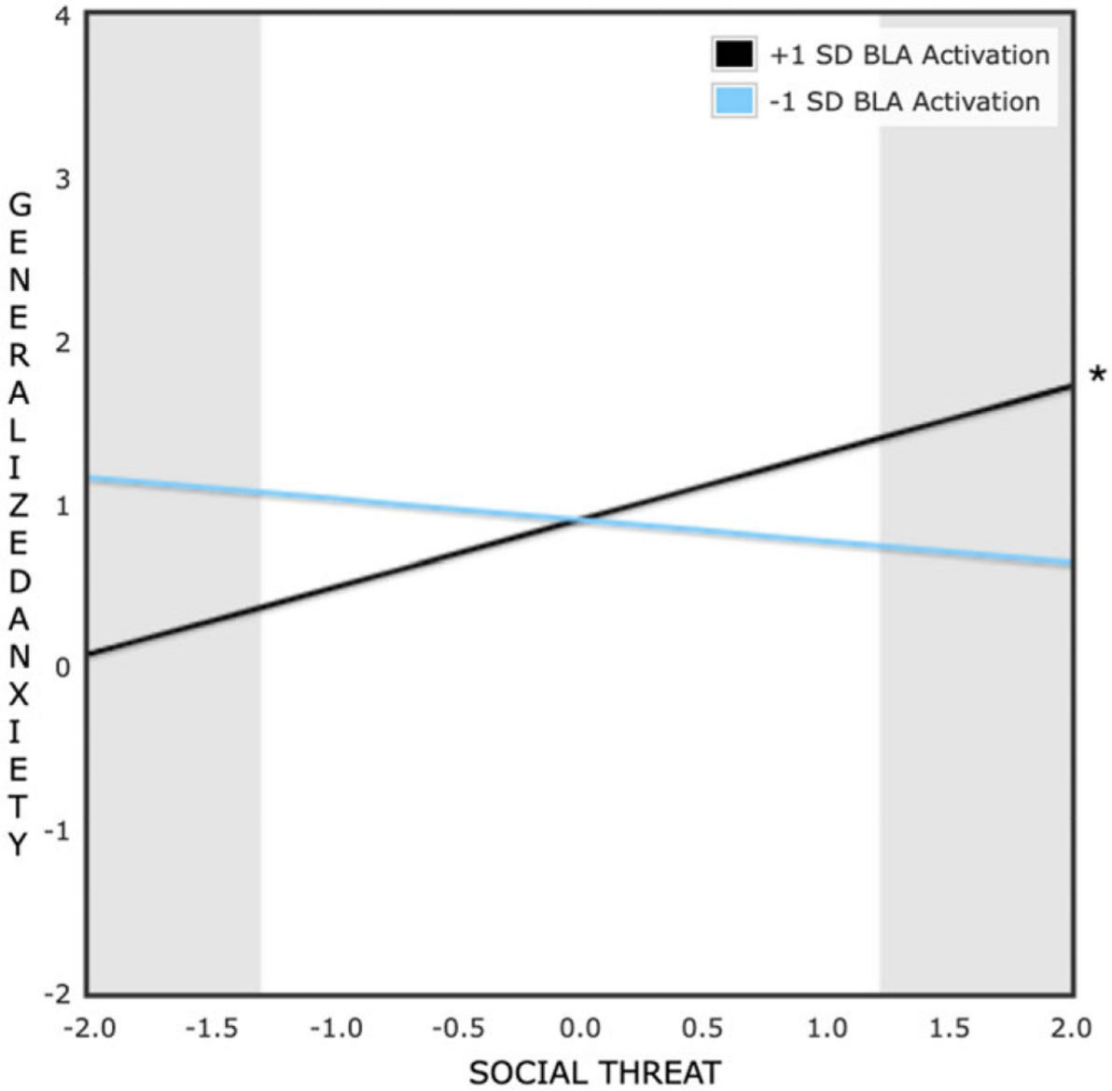
Effect of daily social threat perceptions on two-year follow-up generalized anxiety symptoms at high (+1 SD) and low (−1 SD) levels of bilateral basolateral amygdala activation to social reward (vs. neutral) cues at baseline. Predictors were standardized prior to the formation of this plot; social threat scores are plotted from −2 SD to 2 SD with a mean of 0. *p < .05.

The full structural model (including the social reward factor^1^) was first estimated without the interaction term to examine model fit. Models included 105 participants with 48 free parameters; 24 participants were not included because of missing daily social threat data, which could not be estimated in the full model due to its interaction with the latent factor. To better compare models with and without the interaction term, these participants were also excluded from the model without the interaction term. Again, with eight ROIs loading on the social reward factor, these models were underpowered, and findings are considered tentative. The restricted model (without the interaction term) showed good fit to the data (*χ*^2^ = 62.64, *df* = 56, *p* = .253; RMSEA = .034, CFI = .98, TLI = .98, SRMR = .073; BIC = 5217.60). In this model, significant main effects were seen for baseline social anxiety symptom severity (*B* = .52, SE(B) = .14, *p* < .001) and number of negative peer interactions (B = −.42, SE(B) = .19, *p* = .029). Controlling for all other variables in the model, girls with higher social anxiety symptom severity at baseline and less frequent negative peer interactions at baseline had more severe social anxiety symptoms at two-year follow-up. No significant main effects emerged for daily social threat (*B* = 2.51, SE(B) = 2.41, *p* = .297), pubertal status (*B* = −1.15, SE(B) = 1.90, *p* = .544), or the neural social reward latent factor (*B* = 3.32, SE(B) = 4.11, *p* = .419). The interaction between daily social threat (centered) and the neural social reward factor was then added to the model. Results from the Satorra-Bentler scaled chi-square difference test (chi-square difference score = .05, *df* = 1, *p* > .95) and examination of the BIC (BIC = 5222.30, ΔBIC=+4.70) suggested that adding the interaction term did not significantly improve model fit. Unsurprisingly, then, the interaction term was not significant (B = −.02, SE(B) = 3.87, *p* = .996). Similar to the restricted model, the only significant predictors of two-year social anxiety symptom severity were baseline social anxiety symptom severity (*p* < .001) and number of negative peer interactions (*p* = .030). A visual representation of findings (Figure 2S) can be found in the online supplement.

### Secondary analysis: BLA activity

In line with our initial analytic plan, a secondary analysis tested the interaction between neural activity and daily social threat specifically for bilateral BLA activation to social reward vs. neutral cues, as this was the only region not included in the neural social reward latent factor. FIML was implemented by estimating the variances for the observed exogenous variables in the Model command. The MLR estimator was retained given non-normal distribution of BLA activity. Daily social threat and BLA activity were centered prior to the creation of the interaction term in Mplus.

In this just-identified (*df* = 0) path model (*N* = 129, 35 free parameters; model *R*^2^ = .24, SE = .08, *p* = .002), the interaction between BLA activation to social reward (vs. neutral) cues and daily social threat was significant (*β* = .24; *B* = 4.08, SE(B) = 1.71, *p* = .017). Including this interaction in the model explained an additional 5% of the variance in social anxiety symptom severity (without the interaction term, model *R*^2^ = .19, SE = .07, *p* = .007). As displayed in Table 2, main effects of baseline social anxiety symptoms (*β* = .33; *B* = .43, SE(B) = .13, *p* = .001) and number of negative peer interactions (*β* = −.20; B = −.47, SE(B) = .19, *p* = .016) on two-year social anxiety symptom severity were also seen. A significant main effect of BLA activation emerged (*β* = .20; *B* = 2.71, SE(B) = 1.36, *p* = .047) but is not interpreted due to the presence of a significant interaction. Removing the interaction term from the model, BLA activation had a moderate but non-significant effect on Wave 2 social anxiety symptoms (*β* = .22; *B* = 2.85, SE(B) = 1.58, *p* = .070).

The interaction between daily social threat and BLA activation to social reward (vs. neutral) cues was probed using simple slopes analysis in Mplus. This analysis revealed that the simple slope for the effect of daily social threat on two-year follow-up social anxiety severity was only significant at high levels (+1 SD) of BLA activation to social reward vs. neutral cues (*B* = 11.30, SE = 4.68, *p* = .016; Table 3). Johnson-Neyman analysis in Mplus further showed that the effect of daily social threat on two-year social anxiety symptom severity was significant only at BLA values above .30 SDs above the mean. For reference, one-third of the participants (*n* = 29) had BLA values above .30 SDs above the mean.

Regions of significance on X tests were then conducted. Figure 1 shows the association between daily social threat and social anxiety symptoms moderated by low (−1SD) and high (+1SD) levels of BLA activation to social reward (vs. neutral) cues. The area shaded in gray refers to regions where the two slopes are significantly different. As shown in Figure 1, no significant crossover interaction emerged; only at high levels of social threat were BLA activity and social anxiety symptoms significantly associated.

The interaction between BLA activation to social reward vs. neutral cues and daily social threat predicting total social anxiety symptoms remained significant when the sample was restricted to only participants with social anxiety symptoms collected prior to the COVID-19 pandemic (*N* = 99; interaction *B* = 7.03, SE = 2.94, *p* = .017). The interaction between BLA activation and daily social threat also remained significant when running the model using listwise deletion (interaction *B* = 3.37, SE = 1.37, *p* = .014), which restricted the sample to *N* = 68 with full data.

#### Specificity to social anxiety symptoms

Interactions between BLA activity and daily social threat significantly predicted self-reported generalized anxiety symptoms (interaction *β* = .27, *B* = .72, SE(B) = .25, *p* = .004; model *R*^2^ = .19, SE = .07, *p* = .005) and depressive symptoms (interaction *β* = .25, *B* = 1.94, SE(B) = .84, *p* = .022; model *R*^2^ = .35, SE = .09, *p* < .001) at two-year follow-up (adjusting for baseline symptoms, pubertal status, and number of interactions). Johnson-Neyman analyses revealed that the effect of daily social threat on two-year follow-up generalized anxiety symptoms was significant when BLA activity was above .3 SDs above the mean. The effect of daily social threat on two-year depressive symptoms was significant when BLA activity was above .6 SDs below the mean. Full model results can be found in Table 2.

**Figure 3.**
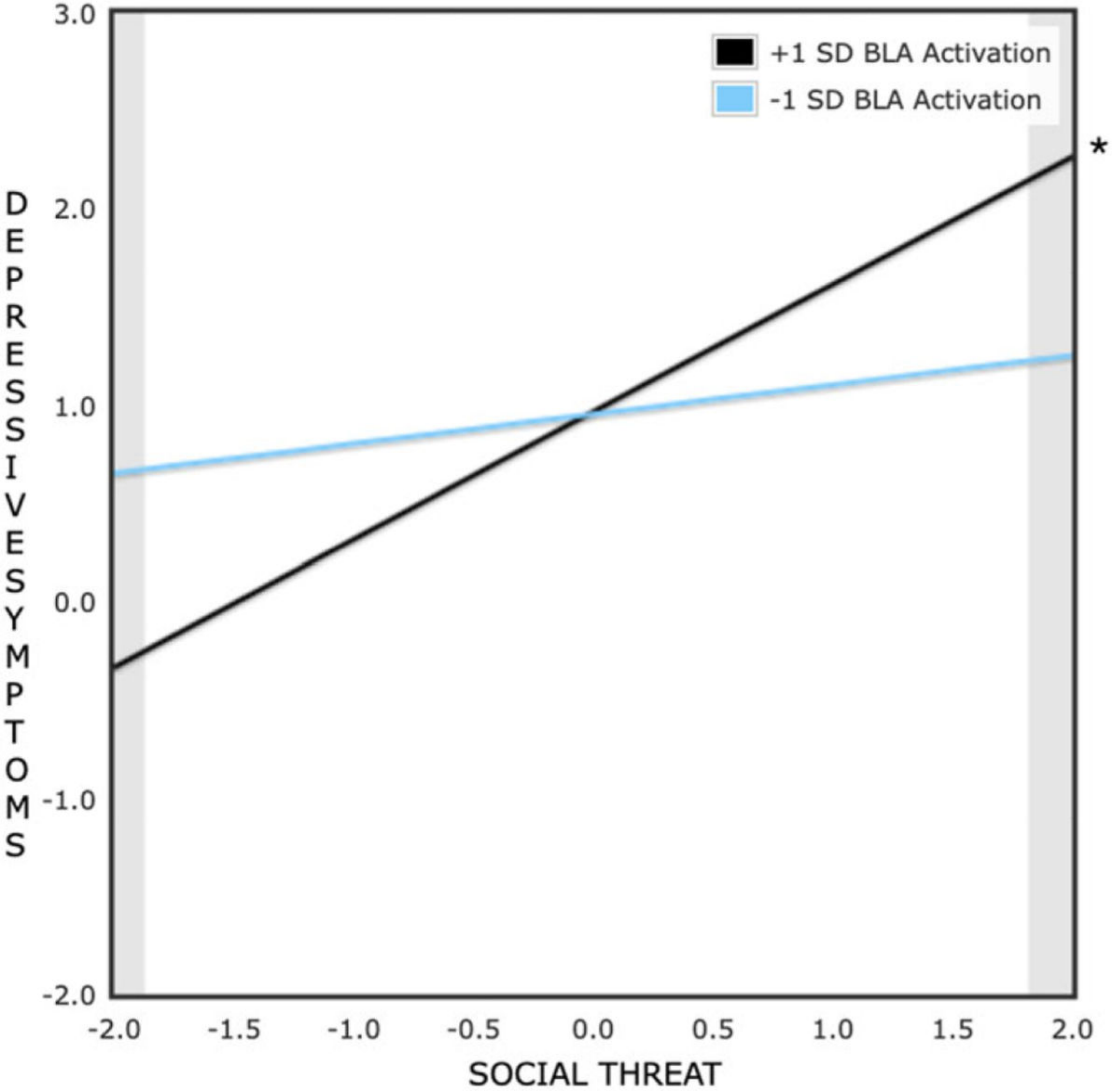
Effect of daily social threat perceptions on two-year follow-up depressive symptoms at high (+1 SD) and low (−1 SD) levels of bilateral basolateral amygdala activation to social reward (vs. neutral) cues at baseline. Predictors were standardized prior to the formation of this plot; social threat scores are plotted from −2 SD to 2 SD with a mean of 0. *p < .05.

Significant crossover interactions emerged for both generalized anxiety symptom and depressive symptom models; at both high and low levels of daily social threat (within the range of −2 SD to + 2 SD), significant differences between low (−1 SD) and high (+1 SD) BLA activity were found (Figs. 2 and 3). The PoI was .50 for the generalized anxiety symptom model and .51 for the depressive symptom model, which supports a differential susceptibility model. Moreover, in these models, BLA reactivity to social reward (vs. neutral) feedback, the suggested susceptibility factor, was not significantly associated with Wave 2 generalized anxiety or depressive symptoms (estimated *rs* < .02, *ps* > .87) or daily social threat (estimated |*r*|s < .17, *ps* > .15), which are necessary conditions to support a differential susceptibility hypothesis (Belsky et al., 2007).

### Specificity to social reward

BLA activation to social reward vs. neutral cues and BLA activation to social punishment vs. neutral cues were significantly correlated (*r* = .53, *p* < .001). However, no interaction emerged between BLA activation to social punishment vs. neutral cues and daily social threat (*β* = .05; *B* = 1.10, SE(*B*) = 2.15, *p* = .607). Additionally, no main effect of daily social threat was found (*β* = −.02; B = −.55, SE(*B*) = 2.48, *p* = .824), though a significant main effect of BLA activation to social punishment vs. neutral cues was seen (*β* = .25; *B* = 3.78, SE(*B*) = 1.63, *p* = .020). A significant main effect of baseline social anxiety symptom severity also emerged (*β* = .34; *B* = .45, SE(*B*) = .13, *p* < .001).

#### Exploratory analysis: NAcc

Though not pre-specified in our initial analytic plan, we conducted an exploratory analysis testing the social anxiety model with the bilateral NAcc ROI (i.e., NAcc activation to social reward vs. neutral cues interacting with daily perceived social threat to predict two-year social anxiety symptoms, adjusting for baseline social anxiety symptoms, pubertal status, and number of negative interactions), given the key role of the NAcc in reward processing. This exploratory analysis allowed us to test whether BLA findings may replicate for another reward-related region in a well-powered model. No significant interaction emerged between bilateral NAcc activation to social reward vs. neutral cues and daily social threat on two-year social anxiety symptoms (*β* = .16; *B* = 2.27, SE(B) = 1.57, *p* = .147); additionally, no main effect of NAcc activation to social reward vs. neutral cues on two-year social anxiety symptoms was found (*β* = .08; *B* = .83, SE = 1.24, *p* = .503).

## Discussion

The present study took a multimethod approach to examine whether heightened neural reactivity to social reward cues places girls at highest risk for social anxiety symptoms when girls perceive high social threat from peers and places girls at lowest risk for social anxiety symptoms when they perceive low social threat from peers. Primary analyses incorporating a neural social reward latent factor failed to support our hypothesized model, though these analyses were underpowered and are thus presented as tentative. Findings from secondary analyses partially supported hypotheses, such that girls with higher bilateral basolateral amygdala (BLA) activation to social reward vs. neutral cues at baseline showed significant associations between daily social threat at baseline and social anxiety symptoms two years later. BLA findings were specific to neural responsivity to social reward (vs. neutral) cues and did not extend to social punishment (vs. neutral) cues. However, the interaction between BLA activity and daily social threat predicted both generalized anxiety symptoms and depressive symptoms.

### BLA activation to social reward cues

In a secondary analysis, bilateral BLA activation to social reward (vs. neutral) cues significantly interacted with daily social threat to predict social anxiety symptom severity two years later. A significant positive association between daily social threat and two-year social anxiety severity emerged only for girls with high BLA activity to social reward (vs. neutral) cues. No significant crossover interaction was observed; at low levels of daily social threat, social anxiety symptoms were relatively low regardless of BLA activity. Only at high levels of social threat were differences in BLA activity associated with differences in social anxiety symptoms, such that girls with high levels of BLA activity exhibited more severe symptoms than girls with low levels of BLA activity.

Conceptually, the BLA is a hub of the salience network and may play a key role in detection processes (Fox et al., 2023). Social reward cues may be particularly salient for the current sample, as early adolescent girls tend to be highly sensitive to social evaluative cues and feedback (Rudolph & Conley, 2005), which is believed to be supported by developmental changes and maturation in brain regions and circuits, including the amygdala (Scherf et al., 2013). The BLA also activates in response to reward-predictive cues during learning to support learning from reward contingencies, particularly through interactions with the orbitofrontal cortex (Lichtenberg et al., 2017; Murray, 2007; Wassum et al., 2011, 2015). Further, this region plays a role in learning and memory through connections with the hippocampus (Yang & Wang, 2017) and in associative learning and motivation through connections with the Nacc (Phillips et al., 2003; Wassum & Izquierdo, 2015). Interactions between high BLA activity and high perceived social threat could lead individuals to expect more negative social feedback in their environments through altered reinforcement learning processes, contributing to more severe social anxiety symptoms over time. High sensitivity to contingencies in the environment that have a high potential to be socially rewarding, such as waiting to be asked to dance, may be detrimental when time and time again, a teen is not asked to dance and feels rejected. Over time, this teen may come to associate potentially rewarding situations with failure, and come to avoid such events, contributing to increases in social anxiety. This interpretation aligns well with Richey et al.’s (2019) Sensitivity Shift Theory. It must be noted, however, that the P-SID task is not a reinforcement learning task, thus interpreting present findings in the context of reward learning is speculative.

Significant findings for the BLA (and not the latent factor) could be attributed to fewer parameters being estimated in the former model (and thus greater power to detect a significant effect), rather than a meaningful difference between the BLA and other regions making up the social reward factor. Notably, however, we did not find a significant interaction between bilateral NAcc activation to social reward vs. neutral cues and perceived social threat on social anxiety symptoms, which could argue against this interpretation. While there are benefits of a latent variable approach, there are also important drawbacks. One potential reason why this approach has not been implemented more often in fMRI research is that latent variable models require large samples; SEM incurs high degrees of freedom due to the high number of parameters. Such large samples are not often seen in fMRI studies (Poldrack et al., 2017), largely due to high costs of running MRI scans. In the present study, unexpected data loss contributed to an underpowered structural equation model; thus, results for the structural model are presented as tentative and should be interpreted with caution. Moreover, as in similar studies (e.g., Baskin-Sommers et al., 2016; Oshri et al., 2019), the small sample precluded our ability to split the sample to run an EFA and CFA on the fMRI data in separate groups, which would be the ideal approach. The social reward factor provided a means to summarize the fMRI data but would need to be confirmed in other samples to speak to generalizability.

Though a crossover interaction was originally hypothesized, BLA findings failed to align with such a differential susceptibility or neurobiological susceptibility to social context model. Importantly, though, the measures used for this study did not allow for an ideal test of these models. Specifically, low levels of perceived social threat do not necessarily imply high levels of perceived social reward. It is still possible that girls with high levels of BLA activity would thrive in environments in which they perceive high levels of social reward (e.g., feeling socially accepted, high frequency of positive social feedback), as they may learn to expect positive social feedback over time. Relatedly, the daily social threat measure is not an objective measure of peer rejection; rather, it is a measure of emotional reactivity in negative social situations, tapping youths’ perceptions of how socially threatened (e.g., criticized, embarrassed, rejected) they feel in negative interactions with peers. While low levels of daily social threat could represent a positive social environment, very low levels of social threat could also index blunted reactivity in negative social situations and/or low awareness of social cues and social experiences. Girls who are relatively unaffected by negative peer interactions or show low social awareness are unlikely to be highly socially anxious, as social anxiety is marked by an intense and persistent fear of negative evaluation. Thus, girls reporting low levels of daily social threat may be at the lowest risk for social anxiety symptoms regardless of brain activity, which could help explain the present pattern of findings.

BLA findings could align with a diathesis-stress model (e.g., Monroe & Simons, 1991), which focuses on how stressful environments exacerbate negative outcomes for individuals with certain underlying vulnerabilities (or diatheses). Interestingly, previous findings from a similar study by Jacho et al., (2019) were also interpreted in the context of a diathesis-stress model, such that neural responsivity to unexpected positive social feedback exacerbated the link between peer victimization and social anxiety symptoms in youth showing high levels of early childhood wariness. In the present study, the small-moderate main effect of BLA reactivity to social reward vs. neutral cues on social anxiety symptoms is also consistent with a diathesis-stress model, as it suggests that high BLA reactivity to reward cues could itself be a risk factor for social anxiety in this sample, but even more so in the presence of high perceived social threat. It is important to note, however, that high neural responses to reward cues are unlikely to be vulnerability factors for most individuals. Indeed, higher neural responses to reward anticipation are thought to underlie higher motivation and pleasure, and are likely protective against the development of depression in adolescence (Keren et al., 2018; Olino et al., 2014), highlighting the nuance required in these interpretations.

### Model specificity: extension to generalized anxiety, depression, and punishment

Contrary to hypotheses, specificity to social anxiety symptoms in the secondary analyses was not supported. Interactions between BLA activation to social reward cues and daily social threat predicted both generalized anxiety symptoms and depressive symptoms at two-year follow-up. Only at moderate to high levels of BLA activity did significant positive associations between daily social threat and generalized anxiety or depressive symptoms emerge. Thus, the interaction between neural social reward function and social threat may be relevant for disorders broadly associated with altered functioning in the positive valence system domain and may help us understand deficits in positive affect, anhedonia, and motivation seen transdiagnostically. Moreover, interactions between neural reward function and daily social threat may contribute to general distress common across internalizing disorders. Including more transdiagnostic outcomes in future work will be valuable.

Interestingly, crossover interactions between BLA activity and daily social threat were observed for both generalized anxiety and depressive symptom models, though for the depression model, significant differences in BLA activity were only seen at extremely high and low levels of daily social threat, calling into question the meaningfulness of this crossover interaction. Findings for the generalized anxiety and depression models could be interpreted in the context of a differential susceptibility model, which assumes that low social threat indicates a more positive social environment, which may be the case. However, it could also be that case that very low levels of perceived social threat indicate lower social awareness, as previously discussed in the context of social anxiety. Moreover, while low social awareness may be protective against social anxiety, this may not be the case for depression or generalized anxiety. For example, girls with lower social awareness may have difficulty making friends and “fitting in”, which could contribute to loneliness and sadness, as well as anxiety around not fitting in. This could be particularly problematic in combination with reduced BLA reactivity to social reward cues, as this pattern of brain activity could impede learning from any positive social interactions that do occur, potentially contributing to lower motivation to seek out socially rewarding experiences and higher social anhedonia. Thus, at low levels of daily social threat, girls with reduced BLA reactivity to social reward cues may be more socially disengaged and disconnected, and therefore at higher risk for depression and generalized anxiety, than youth with increased BLA activity. These potential explanations, though speculative, underscore the nuance needed when interpreting (what may appear to be) differential susceptibility models.

Finally, different patterns of findings for the social anxiety versus generalized anxiety and depression models could be related to differences in measurement of anxiety and depression symptoms. Generalized anxiety and depressive symptoms were self-reported and social anxiety symptoms were clinician-rated. Daily social threat scores were significantly correlated with Wave 1 and Wave 2 depressive symptoms and generalized anxiety symptoms, but were not significantly correlated with Wave 1 or 2 social anxiety symptoms, suggesting that shared method variance could play a role in biasing the generalized anxiety and depressive symptom models. It should also be noted that the present study employed a sample of girls at temperamental risk for social anxiety specifically; it is unclear whether and how present findings are related to the unique nature of this sample and how findings might generalize to girls not recruited on the basis of risk for social anxiety disorder.

Of note, a main effect of BLA activation to social punishment (vs. neutral) cues on two-year follow-up social anxiety symptoms was found, contributing to a large literature showing that heightened amygdala reactivity to potential threat may be one risk factor for the development of anxiety disorders (Shackman et al., 2016). However, interaction effects were specific to BLA activation to social reward cues, and the full model did not replicate for BLA activation to social punishment cues. As this sensitivity analysis was conducted to examine the extent to which brain regions chosen specifically for their role in social reward processing might also be involved in processing social threat cues (and thus may be more accurately described as processing motivationally salient cues), we did not extract separate ROIs for the social punishment condition. Future research might test the current model using social threat-specific ROIs.

### Strengths, limitations, and future directions

Using EMA to measure perceptions of daily social threat was a main strength of this study, with several associated drawbacks. As mentioned, there are different potential ways to interpret this social threat measure. For example, while higher daily social threat scores could be capturing more severe social stress in daily life, girls with higher social threat scores could also be perceiving even relatively neutral interactions with peers as very threatening. Including a more objective measure of peer stress (e.g., from sociometric ratings) in future work testing the proposed model could provide more context with which to interpret present findings. However, this should not discount the value of the present social threat measure, which provides an important indicator of how threatening each individual perceives their social environment to be. In many ways, youths’ perceptions of social threat may be more meaningful to their development than an objective measure. Perhaps more importantly, though, this EMA measure was not well-suited to test a possible differential susceptibility model, as a low social threat score is not a reliable indicator of a positive social context, as previously discussed. Moreover, perceiving some social threat in negative interactions with peers may actually be adaptive and helpful for adolescents tasked with navigating complex social environments. A more ideal test of differential susceptibility would include predictor and outcome variables that have values ranging from truly positive on one end to truly negative on the other end.

Additionally, present analyses and interpretations assume that BLA activity is trait-like, at least in the short term. Concerns have been raised regarding the stability of neural activity, particularly when neural activity is assessed using contrasts (e.g., social reward > neutral; Elliott et al., 2020), and reliable brain-behavior correlations may require thousands of participants (Marek et al., 2022). Integrating within-person changes in brain activity and social threat may help improve reliability in future work. Moreover, as neural activity and social threat were only measured at one time point, it remains unknown whether and how these factors influence each other over time to contribute to social anxiety. Though we speculate on the role of reward learning, the mechanisms through which perceived social threat and brain activity interact to support increases in social anxiety symptoms during adolescence remain to be investigated in future research.

A further strength of the study was recruiting a sample enriched for risk for future social anxiety based on shy and fearful temperament in early adolescence. Though we present preliminary findings comparing key variables between girls high in shy/fearful temperament and girls lower in shy/fearful temperament, we caution against over-interpreting these findings, as with uneven group sizes, this study was not designed for primary group analysis. Finally, for one in five participants, social anxiety symptoms were assessed during the COVID-19 pandemic, a period of time that may have had meaningful impacts on their trajectories of social anxiety symptom development. The stress of the pandemic, decreased opportunities for in-person social exposure, and transitions to novel means of social interaction (e.g., Zoom) could have contributed to increases in social anxiety in girls. Alternatively, though, the pandemic could have contributed to short-term decreases in social anxiety, as many girls no longer had to participate in social interactions that made them nervous (e.g., sports, clubs, eating in front of others). For the current sample, it is difficult to know how the COVID-19 pandemic influenced the developmental trajectories of social anxiety symptoms. However, controlling for COVID-19 group (i.e., data collected pre-pandemic or collected during the pandemic) or restricting the sample to only girls with data collected pre-pandemic did not change the pattern of findings. Future researchers will need to think critically about how to incorporate the COVID-19 pandemic and associated stressors into our understanding of adolescent socioemotional development.

Despite existing limitations, the present study builds on emerging research suggesting that reward processing should not be left out of research on anxiety disorders. Research should continue exploring the role of neural reward function and other constructs associated with reward processing (e.g., behavioral approach) in social and generalized anxiety symptoms (Sequeira et al., 2022). This research should pay close attention to how such constructs are related to anxiety symptoms separate from co-occurring depressive symptoms. Future research could also integrate reward learning into this model, to test whether reward learning is indeed a mechanism contributing to increases in social anxiety symptoms during adolescence. Further, future work should build upon current findings regarding the BLA without discounting other potential brain regions and circuits that may also interact with social threat perceptions to predict social anxiety symptoms in adolescence. Finally, this model should be tested with different developmental periods and with adolescents varying in racial, ethnic, gender, and sexual identities.

## Supplementary Material

1

## Figures and Tables

**Table 1.** Key demographic and clinical characteristics of the sample

	*n* (%)	Mean (SD)	Range
**Wave 1 (Baseline)**			
Age		12.27 (.80)	11–13
Pubertal status (average score)		3.48 (1.05)	1–5
Total family income		7.07 (3.19)	0–10
*Diagnosis (Current)*			
Specific phobia	21 (16.3%)		
Attention-deficit/hyperactivity disorder			
Predominately inattentive	3 (2.3%)		
Combined type	3 (2.3%)		
Unspecified	1 (.8%)		
Oppositional defiant disorder	6 (4.7%)		
Unspecified disruptive behavior disorder	1 (.8%)		
Tic disorder	2 (1.6%)		
Enuresis	2 (1.6%)		
**Wave 2 (Two-Year Follow-Up)**			
Age		14.29 (.81)	13–16
Pubertal status (average score)		4.38 (.73)	1–5
Total family income		7.32 (3.12)	0–10
*Diagnosis (Current)*			
Major depressive disorder	3 (2.3%)		
Persistent depressive disorder	1 (.8%)		
Anxiety disorders			
Social anxiety disorder	23 (17.8%)		
Generalized anxiety disorder	10 (7.8%)		
Specific phobia	13 (10.1%)		
Panic disorder	1 (.8%)		
Separation anxiety disorder	1 (.8%)		
Unspecified anxiety disorder	1 (.8%)		
Obsessive-compulsive disorder	2 (1.6%)		
Post-traumatic stress disorder	1 (.8%)		
Attention-deficit/hyperactivity disorder			
Predominately inattentive	1 (.8%)		
Combined type	1 (.8%)		
Unspecified	1 (.8%)		
Oppositional defiant disorder	3 (2.3%)		
Unspecified disruptive behavior disorder	11 (8.5%)		
*Diagnosis (Past; i.e., between Wave 1 and Wave 2)*			
Major depressive disorder	12 (9.3%)		
Adjustment disorder with depressed mood	2 (1.6%)		
Unspecified depressive disorder	9 (7.0%)		
Anxiety disorders			
Social anxiety disorder	2 (1.6%)		
Generalized anxiety disorder	1 (.8%)		
Specific phobia	4 (3.1%)		
Unspecified anxiety disorder	1 (.8%)		
Oppositional defiant disorder	2 (1.6%)		
*Race/Ethnicity*			
White	87 (67.4%)		
Black/African-American	26 (20.2%)		
Asian	2 (1.6%)		
Biracial	12 (9.3%)		
Native American	1 (.8%)		
Other	1 (.8%)		
Hispanic or Latino	10 (7.8%)		

*Note.* Pubertal status was coded as a continuous variable from 1 (low) to 5 (high); Total family income was reported on a scale of 0–10 in increments of $10,000 (e.g., 0 = $0–10,000, 1 = $10,001–20,000 ... 10 = $100,001+).

**Table 2.** Full results for bilateral basolateral amygdala (BLA) activation to social reward (vs. Neutral) cues and perceived daily social threat predicting social anxiety, generalized anxiety, and depressive symptoms

	*β*	B	SE(B)	*p* − value (*B*)
**Model DV: Social Anxiety Symptoms**				
Intercept	1.53	36.23	8.68	< .001
Pubertal status	– .13	– 2.93	1.83	.108
Baseline social anxiety severity	.33	.43	.13	.001
Number of negative peer interactions	– .20	– .47	.19	.016
Daily social threat perceptions	.13	2.53	1.58	.115
BLA reactivity to social reward (vs. neutral) cues	.20	1.36	1.99	.047
**BLA reactivity X daily social threat**	**.24**	**4.08**	**1.71**	**.017**
Simple slope at low levels of BLA activity (–1 SD)		– 4.97	3.61	.169
Simple slope at moderate levels of BLA activity (Mean)		3.99	2.53	.115
**Simple slope at high levels of BLA activity (+1 SD)**		**12.96**	**5.30**	**.014**
**Model DV: Generalized Anxiety Symptoms**				

Intercept	.90	3.39	1.19	.004
Pubertal status	– .01	– .02	.27	.947
Baseline generalized anxiety severity	.27	.31	.11	.006
Number of negative peer interactions	.07	.03	.03	.350
Daily social threat perceptions	.14	.68	.47	.146
BLA reactivity to social reward (vs. neutral) cues	.00	.00	.22	.998
**BLA reactivity X daily social threat**	**.27**	**.72**	**.25**	**.004**

Simple slope at low levels of BLA activity (–1 SD)		– .87	.73	.235
Simple slope at moderate levels of BLA activity (Mean)		.68	.47	.146
**Simple slope at high levels of BLA activity (+1 SD)**		**2.23**	**.68**	**.001**
**Model DV: Depressive Symptoms**				
Intercept	.82	8.95	4.41	.042
Pubertal status	– .07	– .69	.83	.982
Baseline depressive severity	.28	.43	.14	.003
Number of negative peer interactions	.00	.00	.11	.982
Daily social threat perceptions	.40	5.38	1.67	.001
BLA reactivity to social reward (vs. neutral) cues	.01	.03	.59	.955
**BLA reactivity X daily social threat**	**.25**	**1.94**	**.84**	**.022**
Simple slope at low levels of BLA activity (–1 SD)		1.21	2.02	.549
**Simple slope at moderate levels of BLA activity (Mean)**		5.38	**1.67**	**.001**
**Simple slope at high levels of BLA activity (+1 SD)**		9.55	**2.84**	**.001**

*Note*. Not depicted in this table are means, variances, and correlations of and between independent variables, which were estimated in the model and contribute to the total number of free parameters (*N* = 35 for each model). DV = dependent variable.

## Footnotes

1 Due to limitations regarding the latent factor, including concerns regarding power, we conducted an exploratory analysis taking a complementary approach to data reduction of the fMRI variables that would conserve power. Specifically, a composite variable of social reward neural function was created by averaging activity across all ROIs (with and without the BLA included). When this variable was included in the full model (i.e., path analysis), no significant interaction between this social reward composite variable and social threat on two-year social anxiety symptoms emerged (with BLA: *β* = .07; *B* = 1.55, SE(B) = 3.21, *p* = .630; without BLA: *β* = .04; B = 0.89, SE(*B*) = 3.15, *p* = .778) and no main effects of the social reward composite variable on two-year social anxiety symptoms emerged (*p*s > .13).
