# Supplementary material for: Pathways to adolescent social anxiety: Testing interactions between neural social reward function and perceived social threat in daily life
Source: Dev Psychopathol. Author manuscript; Available in PMC 2025 Jul 8. (PMC11599470; doi:10.1017/S0954579424001068)
Supplement: 1 [file NIHMS1992808-supplement-1.docx]

**Table 1S. Descriptive Statistics and Intercorrelations Between Observed Variables**

|  | 1 | 2 | 3 | 4 | 5 | 6 | 7 | 8 | 9 | 10 | 11 | 12 | 13 | 14 | 15 | 16 | 17 | 18 | 19 |
| --- | --- | --- | --- | --- | --- | --- | --- | --- | --- | --- | --- | --- | --- | --- | --- | --- | --- | --- | --- |
| 1. Age (Wave 1) | 1 |  |  |  |  |  |  |  |  |  |  |  |  |  |  |  |  |  |  |
| 2. PDS (Wave 1) | **.44** | 1 |  |  |  |  |  |  |  |  |  |  |  |  |  |  |  |  |  |
| 3. Social anxiety sx (Wave 1) | .13 | -.01 | 1 |  |  |  |  |  |  |  |  |  |  |  |  |  |  |  |  |
| 4. Social anxiety sx (Wave 2) | -.08 | -.08 | **.34** | 1 |  |  |  |  |  |  |  |  |  |  |  |  |  |  |  |
| 5. GAD sx (Wave 1) | .08 | .00 | **.60** | **.35** | 1 |  |  |  |  |  |  |  |  |  |  |  |  |  |  |
| 6. GAD sx (Wave 2) | .04 | .04 | .16 | **.48** | **.30** | 1 |  |  |  |  |  |  |  |  |  |  |  |  |  |
| 7. Depressive sx (Wave 1) | -.10 | -.02 | **.37** | **.25** | **.54** | **.25** | 1 |  |  |  |  |  |  |  |  |  |  |  |  |
| 8. Depressive sx (Wave 2) | -.10 | -.02 | .13 | **.31** | **.29** | **.64** | **.41** | 1 |  |  |  |  |  |  |  |  |  |  |  |
| 9. Daily social threat | .00 | .02 | .11 | .09 | **.39** | **.23** | **.28** | **.39** | 1 |  |  |  |  |  |  |  |  |  |  |
| 10. # of negative interactions | **.20** | -.17 | .01 | -.12 | .10 | .13 | -.06 | .07 | **.20** | 1 |  |  |  |  |  |  |  |  |  |
| 11. Anterior insula | -.01 | .15 | **-.26** | .06 | -.14 | .07 | -.14 | .07 | -.19 | -.05 | 1 |  |  |  |  |  |  |  |  |
| 12. Basolateral amygdala | -.10 | -.06 | -.08 | .14 | -.08 | -.07 | .08 | -.06 | -.10 | .04 | **.36** | 1 |  |  |  |  |  |  |  |
| 13. Caudate body | .01 | .14 | -.01 | .03 | .05 | .01 | -.02 | -.03 | -.11 | .04 | **.56** | **.26** | 1 |  |  |  |  |  |  |
| 14. Caudate head | -.09 | .10 | -.07 | .05 | .00 | -.06 | -.03 | -.03 | -.08 | .04 | **.52** | **.25** | **.76** | 1 |  |  |  |  |  |
| 15. Nucleus accumbens | -.10 | .07 | -.05 | .10 | -.10 | .03 | -.10 | -.07 | -.14 | -.15 | **.45** | **.29** | **.41** | **.53** | 1 |  |  |  |  |
| 16. MDN | -.07 | .06 | -.05 | .02 | -.05 | -.05 | -.20 | -.15 | **-.28** | .01 | **.58** | **.22** | **.76** | **.63** | **.42** | 1 |  |  |  |
| 17. Precuneus | -.13 | -.03 | -.04 | .03 | .00 | -.01 | -.05 | -.10 | -.11 | .23 | **.50** | **.37** | **.53** | **.44** | **.24** | **.63** | 1 |  |  |
| 18. Putamen | -.13 | .04 | -.05 | .11 | -.01 | .13 | -.16 | -.01 | -.17 | .01 | **.68** | **.22** | **.76** | **.63** | **.44** | **.80** | **.60** | 1 |  |
| 19. dorsal ACC | .13 | .19 | -.01 | .00 | -.06 | .09 | -.11 | -.02 | -.13 | -.03 | **.84** | **.26** | **.67** | **.55** | **.40** | **.63** | **.53** | **.71** | 1 |
|  |  |  |  |  |  |  |  |  |  |  |  |  |  |  |  |  |  |  |  |
| **Mean** | 12.3 | 3.5 | 24.3 | 28.5 | 4.1 | 4.8 | 9.2 | 10.5 | .98 | 16.2 | .39 | .09 | .51 | .81 | .56 | .79 | .12 | .20 | .27 |
| **SD** | .80 | 1.1 | 18.1 | 23.8 | 3.2 | 3.7 | 7.1 | 11.0 | .79 | 10.1 | 1.7 | 1.8 | 2.0 | 1.9 | 2.2 | 2.2 | 1.7 | 1.6 | 1.8 |
| **Skewness** | .25 | -.34 | .99 | 1.10 | .58 | .53 | .95 | 1.67 | .88 | .73 | -.06 | .96 | .35 | .21 | .39 | -.22 | -.01 | -.22 | .45 |
| **Kurtosis** | -1.0 | -.76 | .51 | .82 | -.39 | -.68 | .30 | 3.37 | .50 | -.39 | .52 | 3.08 | .17 | .55 | .46 | .96 | .68 | -.11 | .44 |

*Note.* Bolded values indicate *p*<.05; activity for all brain regions is for the contrast social reward vs. neutral anticipation; PDS = Pubertal Development Scale, sx = symptoms, GAD = Generalized Anxiety Disorder, MDN = mediodorsal nucleus of the thalamus, ACC = anterior cingulate cortex, SD = standard deviation. Age is included in this table for descriptive purposes but was not used in any analyses.

**Supplemental Results**

**Neural Social Reward Latent Factor**

Eighty-seven participants had usable fMRI data and were included in the EFA. All data were inspected closely prior to the EFA; no regions exhibited skewness values > 2, and only the BLA showed potentially meaningful kurtosis (3.08) due to the presence of two “extreme outliers” (i.e., data points in the third quartile + 3*interquartile range or in the first quartile – 3*interquartile range). There was no reason to assume that these data points were errors in the dataset, thus they were left as is. However, it should be noted that none of the results of the EFA changed when these outliers were winsorized.

All EFA models were estimated in Mplus using an oblique Geomin rotation (the default rotation criterion for EFA) because of its ideal balancing of interpretability and factor complexity (Browne, 2001; Sass & Schmitt, 2010). The optimal number of factors was determined through consideration of a parallel analysis (Horn, 1965) and interpretability of the resulting factors. The eigenvalues of the estimated correlation matrix exceeded the random data generated eigenvalues for only the first factor (first three empirical eigenvalues = 5.26, 0.96, 0.83; first three random data eigenvalues = 1.52, 1.34, 1.21).

Though a one-factor model was suggested, model fit was poor (χ2= 97.78, *df*=27, *p*<.001, RMSEA=.17, CFI=.86, TLI=.82, SRMR=.06). A two-factor solution significantly improved model fit ($\Delta$χ2= 56.0, $\Delta$*df*=8, *p*<.001) but was not indicated by the parallel analysis and was not theoretically indicated; for this solution, the AI loaded on its own factor (1.14) and all other regions loaded significantly (with loadings above .41) on a second factor, with the exception of the BLA, which failed to load significantly on either factor. A three-factor solution failed to converge. Examining factor loadings for the one-factor solution revealed relatively low loading of BLA activity (.32) relative to other variables (NAcc=.51, AI=.76, Caudate Body=.86, Caudate Head=.75, MDN=.86, Precuneus=.66, Putamen=.89, dACC=.81). The decision was made a priori to remove variables with factor loadings below 0.40. Modification indices were also considered to improve model fit; only modifications that would contribute to a chi-square change larger than 10 and were theoretically sound were considered. Modification indices indicated that adding a correlation between the two regions of the caudate would contribute to a chi-square change of 15 and that adding a correlation between the dACC and AI would contribute to a chi-square change of 39. These correlations were sensible from a theoretical standpoint; one would expect that activation in the head and body of the caudate would be highly correlated, and the dACC and AI have strong structural connections and often coactivate functionally in affective salience tasks (Ghaziri et al., 2017).

A confirmatory factor analysis was run on this modified model, which removed the BLA and added correlations added between the dACC and AI, as well as between the two caudate regions. Variance of the NAcc factor was constrained to 1 to fix the scale of the latent factor, as this was chosen a priori as the most representative ROI of the network. Model fit for this final neural social reward latent factor was good (χ2=25.54, *df*=18, *p*=.111; RMSEA=.069, CFI=.99, TLI=.98, SRMR=.038).

**Supplemental References**

Bentler, P. M., & Chou, C. P. (1987). Practical issues in structural modeling. *Sociological Methods & Research*, *16*(1), 78-117.

Browne, M. W. (2001). An overview of analytic rotation in exploratory factor analysis. *Multivariate Behavioral Research*, *36*(1), 111-150.

Ghaziri, J., Tucholka, A., Girard, G., Houde, J. C., Boucher, O., Gilbert, G., ... & Nguyen, D. K. (2017). The corticocortical structural connectivity of the human insula. *Cerebral Cortex*, *27*(2), 1216-1228.

Horn, J. L. (1965). A rationale and test for the number of factors in factor analysis. *Psychometrika*, 30, 179-185.

Sass, D. A., & Schmitt, T. A. (2010). A comparative investigation of rotation criteria within exploratory factor analysis. *Multivariate Behavioral Research*, *45*(1), 73-103.

*.*

**Figure 1S.** Regions-of-interest used in analyses: (A) precuneus (yellow) and dACC (red); (B) BLA;

(C) putamen (green), NAcc (white), caudate body (copper), caudate head (red), AI (blue) (red); (D) MDN (blue).


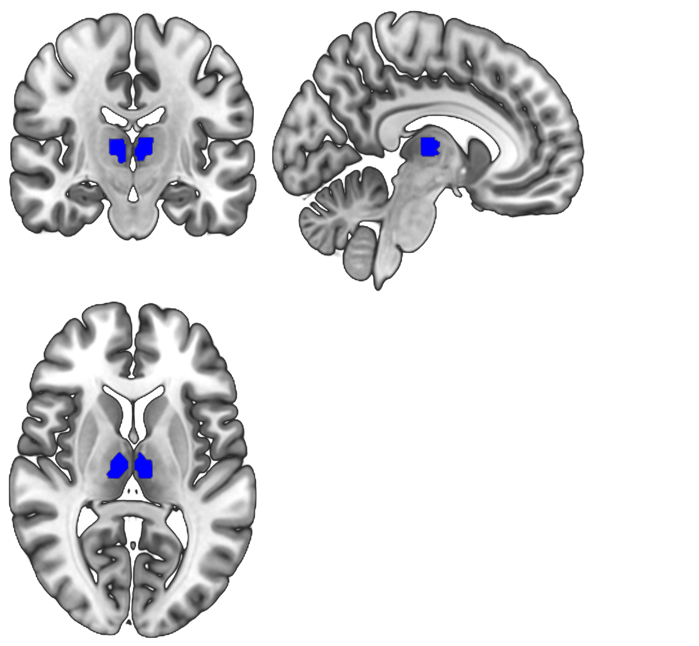

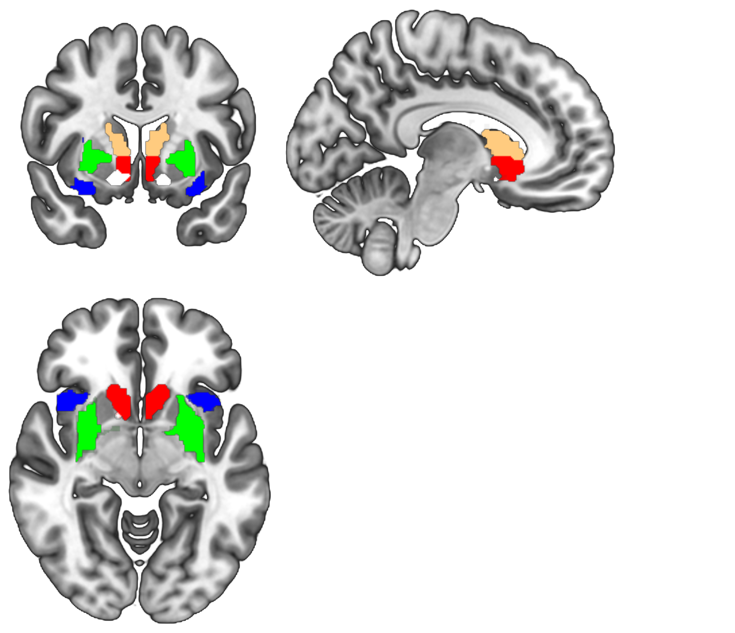

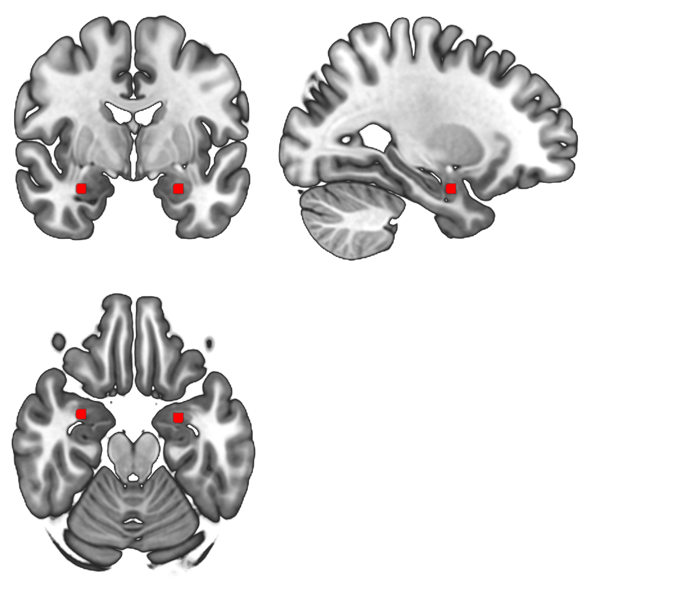

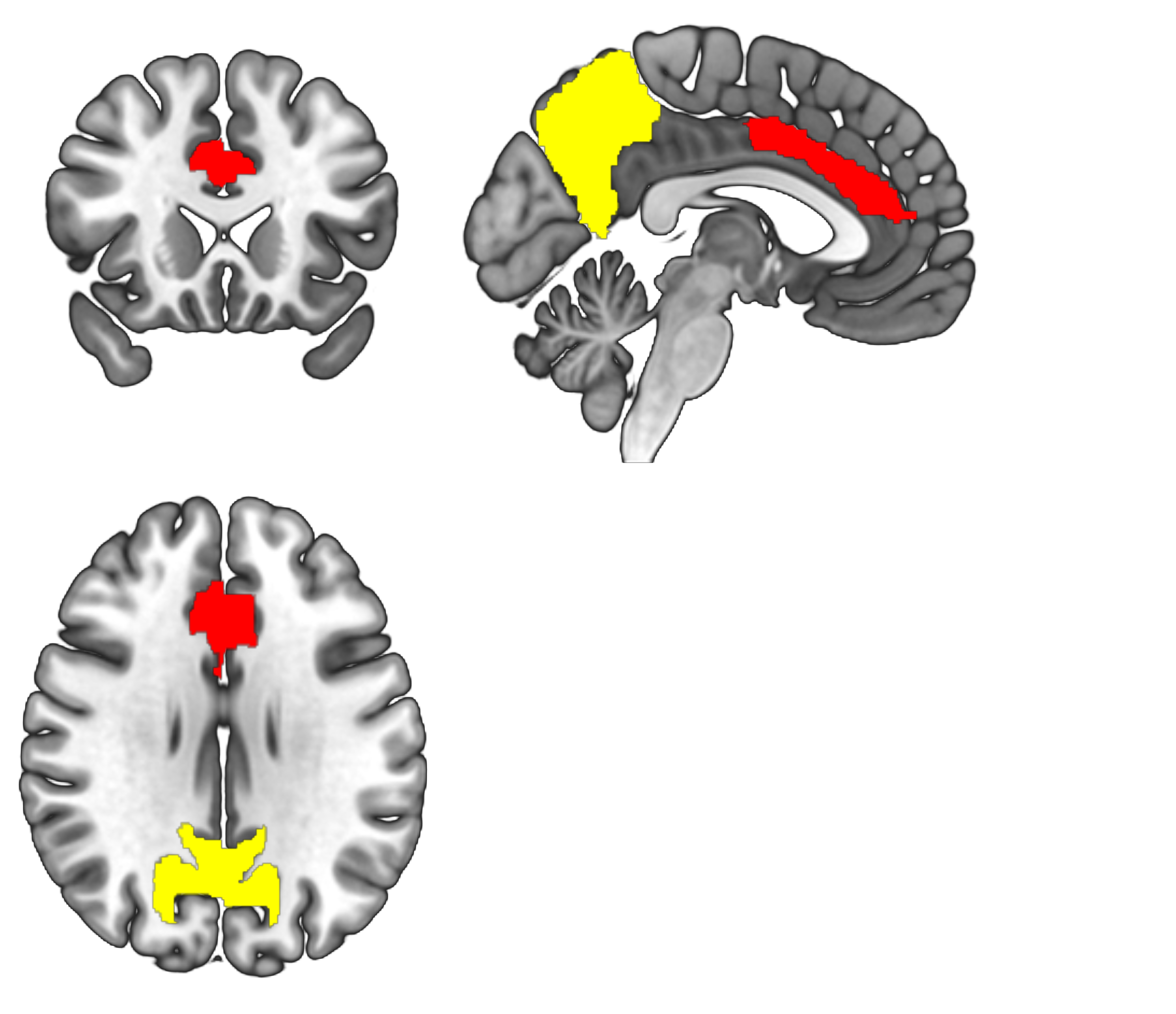


B

A

D

C
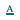


**Figure 2S.** Results from the full structural equation model. Unstandardized estimates and SEs are presented; estimates are bolded at *p*<.05.

Social anxiety symptoms (Wave 2)

Daily social threat

Social anxiety symptoms (Wave 1)

Pubertal status

Number of negative interactions

NAcc

dACC

AI

Putamen

Precuneus

Caudate body

Caudate head

MDN

1.00 (.00)

1.83 (.64)

1.49 (.52)

1.81 (.62)

1.26 (.50)

2.20 (.78)

1.80 (.58)

2.26 (.78)

3.50 (.58)

1.22 (.29)

1.01 (.21)

.51 (.16)

1.60 (.27)

1.16 (.24)

1.61 (.27)

.83 (.16)

-.02 (3.87)

2.51 (2.41)

**-.42 (.19)**

**.52 (.14)**

-1.16 (1.90)

.07 (.08)

.54 (.38)

3.52 (4.46)

490.06 (93.08)

.73 (.21)

.45 (.22)
